# Autophagy-Related *IRGM* Polymorphism Is Associated with Mortality of Patients with Severe Sepsis

**DOI:** 10.1371/journal.pone.0091522

**Published:** 2014-03-13

**Authors:** Tomonori Kimura, Eizo Watanabe, Teruo Sakamoto, Osamu Takasu, Toshiaki Ikeda, Kazumi Ikeda, Joji Kotani, Nobuya Kitamura, Tomohito Sadahiro, Yoshihisa Tateishi, Koichiro Shinozaki, Shigeto Oda

**Affiliations:** 1 Department of Emergency and Critical Care Medicine, Graduate School of Medicine, Chiba University, 1-8-1 Inohana, Chu-ku, Chiba City, Chiba, Japan; 2 Department of Emergency and Critical Care Medicine, Kurume University School of Medicine, 67 Asahi-machi, Kurume City, Fukuoka, Japan; 3 Division of Critical Care and Emergency Medicine, Tokyo Medical University Hachioji Medical Center, 1163 Tate-machi, Hachioji City, Tokyo, Japan; 4 Department of Emergency, Disaster and Critical Care Medicine, Hyogo College of Medicine, 1-1, Mukogawa-cho, Nishinomiya City, Hyogo, Japan; 5 Department of Emergency and Critical Care Medicine, Kimitsu Chuo Hospital, 1010 Sakurai, Kisarazu City, Chiba, Japan; D’or Institute of Research and Education, Brazil

## Abstract

**Objective:**

Autophagy is the regulated catabolic process for recycling damaged or unnecessary organelles, which plays crucial roles in cell survival during nutrient deficiency, and innate immune defense against pathogenic microorganisms. Autophagy has been also reported to be involved in various conditions including inflammatory diseases. *IRGM* (human immunity-related GTPase) has an important function in eliminating *Mycobacterium tuberculosis* from host cells *via* autophagy. We examined the association between genetic polymorphism and clinical course/outcome in severely septic patients.

**Methods:**

The study included 125 patients with severe sepsis/septic shock (SS) and 104 non-sepsis patients who were admitted to the intensive care unit (ICU) of Chiba University Hospital between October 2001 and September 2008 (discovery cohort) and 268 SS patients and 454 non-sepsis patients who were admitted to ICUs of five Japanese institutions including Chiba University Hospital between October 2008 and September 2012 (multi-center validation cohort). Three hundred forty seven healthy volunteers who consented to this study were also included. Genotyping was performed for a single-nucleotide polymorphism (SNP) within the coding region of *IRGM*, IRGM(+313) (rs10065172). Lipopolysaccharide challenge of whole blood from randomly selected healthy volunteers (n = 70) was performed for comparison of *IRGM* mRNA expression among different genotypes.

**Results:**

No significant difference in genotypic distributions (CC/CT/TT) at the IRGM(+313) locus was observed among the three subject groups (SS, non-sepsis, and healthy volunteers) in either cohort. When mortality were compared, no significant difference was observed in the non-sepsis group, while TT homozygotes exhibited a significantly higher mortality than the CC+CT genotype category in the SS group for both cohorts (*P* = 0.043, 0.037). Lipopolysaccharide challenge to whole blood showed a significant suppression of *IRGM* mRNA expression in TT compared with the CC+CT genotype category (*P* = 0.019).

**Conclusions:**

The data suggest that the IRGM(+313), an autophagy-related polymorphic locus, influences outcome in severely septic patients, with the possible involvement of autophagy in sepsis exacerbation.

## Introduction

Severe sepsis is the host inflammatory response to infection with presence of organ dysfunction [1]. It is a critical condition that is very difficult to survive, as indicated by high mortality rates ranging from 30% and 80% in various countries [2], [3]. After overcoming the acute inflammatory response phase and remission of SIRS, septic patients go into an anti-inflammatory phase, which is even more difficult to overcome (CARS; compensatory anti-inflammatory response syndrome). The subsequent development of immunoparalysis is believed to affect the prognosis of sepsis, particularly during the post-acute phase [4]–[6].

Apoptosis (type I programmed cell death) of immune effector cells plays a crucial role in the pathophysiology of CARS and immunoparalysis in sepsis, and the regulation of apoptosis is expected to improve survival in sepsis [7], [8]. On the other hand, the involvement of autophagy (type II programmed cell death) in the pathophysiology of sepsis has attracted the attention of both researchers and clinicians [9]–[11]. Autophagy (“self-eating”), a regulated catabolic process, has roles in the degradation of unnecessary organelles, elimination of pathogenic microorganisms, and tumor suppression [12]. Sepsis-induced organ failure is considered to be the summation of cellular dysfunction induced by cell death of different cells [13], and vital organ failure may be related to immune system abnormalities. Recently, the genome-wide association study (GWAS) approach has enabled systematic searches of disease susceptibility genes. Using this methodology, an autophagy-related gene, autophagy-related protein 16-like 1 (*ATG16L1*), was identified as a disease susceptibility gene closely involved in the pathophysiology of Crohn’s disease, an inflammatory bowel disease [14]. In addition, an association between IRGM (a human immunity-related GTPase) expression and the induction and execution of autophagy upon bacterial infections, such as tuberculosis, has been reported with regulation of autophagy formation in proportion to IRGM expression [15]. Furthermore, genetic polymorphism of *IRGM* has been confirmed to be involved in the development of inflammatory bowel disease and in the induction of autophagy [16].

To investigate how autophagy is involved in the pathophysiology of severe sepsis/septic shock, *i.e.*, a highly systemic inflammatory disease, we examined the association between a single-nucleotide polymorphism (SNP) in the autophagy-related IRGM gene and clinical outcomes in severe sepsis for two cohorts (one single-center cohort and one multi-center cohort) in Japanese population. We also examined the mRNA expression of autophagy-related SNP under stimulus by lipopolysaccharide (LPS) *ex vivo*.

## Materials and Methods

### 1. Discovery Cohort

The protocol was approved by institutional Ethic Committees at all the 5 participating institutes (the Ethics Committee of Chiba University School of Medicine (permission number 205), the Ethical Committee of Kurume University (bioethics permission number 49), the Medical Research Ethics Committee of Tokyo Medical University, the Ethics Review Board of Hyogo College of Medicine, and the Ethics Committee of Kimitsu Chuo Hospital). After approval by the institutional ethics committees, written informed consent from patients or their next of kin was obtained. In discovery cohort, 259 critically ill patients admitted to the general intensive care unit (ICU) of Chiba university hospital in Chiba, Japan, between October 2001 and September 2008. Inclusion criteria were admitted to the ICU, 18 years of age or older, patients capable of obtaining informed consent in writing own, family, or the legal representative. Exclusion criteria included pregnancy, treatment in the hematologic malignancies, patients receiving radiation treatment and chemotherapy, history of genetic therapy, and outside the scope of active treatment. Blood samples were obtained immediately after admission to the ICU. Centrifuged blood samples were kept separate blood cells and serum at −80°C. The genomic DNA was extracted from whole blood cells.

### 2. Multi-center Validation Cohort

In multi-center validation cohort, 793 critically ill patients admitted to the five general ICU including the five tertiary medical centers, or Kurume University Hospital, Tokyo Medical University Hachioji Medical Center, Hyogo College of Medicine, Kimitsu Chuo Hospital and Chiba University Hospital from October 2008 to September 2012. Inclusion and exclusion criteria were the same as the discovery cohort. On admission at the ICUs, blood samples were obtained. The blood cells were refrigerated and collected to Chiba University Hospital, and the genomic DNA was thereafter extracted.

### 3. Data Collection

Baseline characteristics (age, gender), as well as clinical data including length of ICU stay, Sequential Organ failure Assessment (SOFA) scores [17], Acute Physiology and Chronic Health Evaluation (APACHE) II scores [18], morbidity of severe sepsis and septic shock, ICU mortality were obtained after the patients were documented at study entry. The APACHE II scores and SOFA scores were calculated in the first 24 hours after admission.

The diagnosis of systemic inflammatory response syndrome (SIRS) and sepsis, severe sepsis and septic shock were based on the criteria presented at the American College of Chest Physicians/Society of Critical Care Medicine Consensus Conference in 1992 [19]. In the present study, both severe sepsis and septic shock is expressed together as SS group.

### 4. IRGM(+313) (rs10065172) Genotyping

Genomic DNA was extracted from ethylenediaminetetraacetic acid anticoagulated blood using Qiagen’s QIAamp DNA Minikit (Qiagen, Valencia, CA) according to manufacturer’s instructions. We amplified the target region of DNA by polymerase chain reaction (PCR) with primers (Applied Biosystems, Foster City, CA) specific for the sequence of IRGM(+313) (rs10065372), a SNP which presents at the position of +313 inside the exon 2 of *IRGM* in the chromosome 5q33.1 [20], [21]. Each 25 µL of PCR mixture contained 20 ng of genomic DNA, 900 nM primers, 250 nM probes, and 12.5 µL of TaqMan Universal PCR master mix (Applied Biosystems, Foster City, CA), which is a solution containing buffer, uracil-N-glycosylase, deoxyribonucleotides, uridine, passive reference dye (ROX), and TadGold DNA polymerase. Amplification was done under the following conditions: 50°C for 2 min, 95°C for 10 min, and 40 cycles at 92°C for 15 sec and 60°C for 1 min. Fluorescence in each well was measured before and after PCR using an ABI PRISM 7000 Sequence Detection System (Applied Biosystems, Foster City, CA).

### 5. *IRGM* mRNA Expression *ex vivo*

The subjects were 347 healthy volunteers who consented to this study. Extraction of genomic DNA and *IRGM* polymorphisms were analyzed by the method described above.

The randomly selected 70 healthy volunteers, whole blood mixed 10 mL:10 mL with cell culture medium (RPMI 1640 (Wako, Tokyo, Japan)) were transferred to 12-well microtiter plates (Falcon, Mountain View, CA). Samples were incubated at 37°C and 5% CO_2_ with endotoxin (lipopolysaccharide, 1 ng/mL; from *Salmonella friedenau*) for 4 hours. Control mixtures were incubated without endotoxin. After incubation, the supernatants were separated and stored frozen at −80°C. 300 µL of the RNA Protect Cell Reagent (Qiagen, Valencia, CA) was added to the blood cell component after centrifugation, and the mixture was stored at −80°C. Each mRNA was extracted using QIAamp RNA Blood Mini Kit (Qiagen, Valencia, CA) from the blood cells. The mRNA was reversed transcribed into complementary DNA (cDNA) using the High Capacity cDNA Reverse Transcription Kit (Applied Biosystems, Foster City, CA) according to the manufacturer’s instructions. Real-time PCR with specific fluorescence-labeled probe was used to quantify mRNA expression of *IRGM*. The reaction was performed in 96-well microtiter plates with an ABI PRISM 7000 Sequence Detection System. Primers (glyceraldehyde-3-phosphate dehydrogenase; Assay ID: Hs03929097_g1, Applied Biosystems, Foster City, CA) and regents were purchased from Applied Biosystems Inc. PCR reactions were performed in a total volume of 50 µL containing cDNA samples, TaqMan Universal PCR master mix (Applied Biosystems, Foster City, CA). TaqMan glyceraldehyde-3-phosphate dehydrogenase (GAPDH) endogenous control reagent was used as internal control for normalization. Amplification was performed under the following conditions: 50°C for 2 min, 95°C for 10 min, 40 cycles at 95°C for 15 sec, and 60°C for 1 min. Fluorescence in each well was measured using an ABI PRISM 7000 Sequence Detection System. The mRNA expression levels were assessed by relative quantification (RQ). Cycle threshold values for IRGM gene were determined and abundance in comparison to GAPDH was calculated. qRT-PCR results were recorded for IRGM gene as fold change of LPS stimulated vs non-stimulated blood at 4 h using ΔΔC(t) method [22]. PCR products were evaluated by dissociation curves to confirm single amplicons and the absence of significant primer–dimer contamination.

### 6. Statistical Analysis

The Hardy-Weinberg equilibrium for the population distribution of the variant alleles was determined according to the approach described by Guo and Thompson [23]. Allelic *chi*-squares were examined for each SNP and the correlation/trend test was performed. Odds ratios (ORs) were calculated and *P* values were decided with statistical significance defined by *P*≤0.05 using HelixTree™ software (Golden Helix Inc, Bozeman, MT). We considered the differences significant with respect to mortality at a full scan permutation of the correlation/trend test *P*-value of <0.05. Fold change, or ΔRQ, was calculated to determine the magnitude of difference in gene expression and a Mann-Whitney U test was performed to demonstrate the reproducibility of the changes observed in the IRGM gene after LPS challenge. We compared the variables in different groups using the unpaired Student’s t-test and Mann-Whitney U test, depending on the type of variable. Statistical significance was defined as *P*<0.05. Statistical analyses were performed with the GraphPad Prism 5 software package for Windows (GraphPad Software, CA, USA).

## Results

### 1. Baseline Characteristics of Discovery Cohort and Multi-Center Validation Cohort

Table 1 summarizes the baseline characteristics of the discovery cohort (n = 259) and the multi-center validation cohort (n = 793). No significant difference in age, gender, or length of ICU stay was observed between the two cohorts. In addition, no significant difference in SOFA or APACHE II score was detectable between the two cohorts, which indicated that there was no marked difference in patient backgrounds between the two cohorts. The incidence and mortality rates of severe sepsis/septic shock tended to be greater in the discovery cohort, but the difference was not statistically significant.

**Table 1:** Baseline characteristics of the study population.

	Discovery cohort				Multi-center validation cohort			
	All	Non-sepsis patients	SS patients	*P* value	All	Non-sepsis patients	SS patients	*P* value
	n = 259	n = 104	n = 125		n = 793	n = 454	n = 271	
Age (yaers), mean±SD	57±17	57±17	58±17	0.492*	64±17	62±18	67±15	0.0008*
Male/female gender, n	146/113	60/44	69/56	0.659**	517/276	296/158	176/95	0.915**
Length of ICU stay (days), mean±SD	12.5±17.3	6.0±7.5	18.5±22	<0.0001*	17.3±25	12.7±17.4	26.1±34.9	<0.0001*
SOFA score, mean±SD	7.1±5.1	3.8±3.1	10.3±4.6	<0.0001*	6.4±4.4	4.8±3.5	9.5±4.2	<0.0001*
APACHE II score, mean±SD	16.4±9.0	10.6±6.2	21.9±8.3	<0.0001*	17.3±8.8	15.0±8.3	21.8±7.9	<0.0001*
Severe sepsis morbidity (%)	48				33.8			
Mortality (%)	17	5.71	29.6	<0.0001**	12.2	5.4	25.3	<0.0001**
Post-surgical operation								
Post-cardiovascular surgery, n(%)	28 (10.8)	24 (23.0)	4 (3.2)		21 (2.6)	5 (1.1)	12 (4.4)	
Post-gastrointestinal surgery, n(%)	33 (12.7)	11 (10.6)	15 (12.0)		79 (10.0)	25 (5.5)	43 (15.9)	
Others, n(%)	20 (7.7)	12 (11.5)	6 (4.8)		6 (0.8)	3 (0.7)	3 (1.1)	
Intracranial disease (ICH/CI), n(%)	5 (2.0)	0 (0)	4 (3.2)		68 (8.6)	62 (13.7)	3 (1.1)	
Respiratory failure, n(%)	28 (10.8)	4 (3.8)	22 (17.6)		77 (9.7)	15 (3.3)	51 (18.8)	
Heart failure, n(%)	10 (3.9)	4 (3.8)	3 (2.4)		72 (9.1)	61 (13.4)	8 (3.0)	
Endogenous abdominal disease								
Acute pancreatitis, n(%)	22 (8.5)	16 (15.4)	6 (4.8)		39 (4.9)	22 (4.8)	14 (5.2)	
Gastrointestinal bleeding, n(%)	5 (2.0)	2 (1.9)	3 (2.4)		36 (4.5)	33 (7.3)	2 (0.7)	
Hepatic failure, n(%)	7 (2.7)	3 (2.9)	4 (3.2)		19 (2.4)	7 (1.5)	9 (3.3)	
Others, n(%)	13 (5.0)	1 (1.0)	9 (7.2)		34 (4.3)	10 (2.2)	21 (7.7)	
CPAOA, n(%)	4 (1.5)	2 (1.9)	2 (1.6)		39 (4.9)	32 (7.0)	4 (1.5)	
Trauma, n(%)	11 (4.3)	9 (8.7)	0 (0)		119 (15.0)	105 (23.1)	8 (3.0)	
Intoxication, n(%)	8 (3.1)	4 (3.8)	4 (3.2)		23 (2.9)	17 (3.7)	6 (2.2)	
Burn, n(%)	2 (0.8)	1 (1.0)	0 (0)		12 (1.5)	7 (1.5)	4 (1.5)	
Others, n(%)	63 (24.3)	11 (10.6)	43 (34.4)		149 (18.8)	50 (11.0)	83 (30.6)	

*P* values were calculated with Student’s t-test* and Fisher’s exact test**. SS, severe sepsis/septic shock; ICU, intensive care unit; SOFA, sequential organ failure assessment; APACHE II, the acute physiology and chronic health evaluation; ICH, intracerebral hemorrhage; CI, cerebral infarction; CPAOA, cardiopulmonary arrest on arrival; SD, standard deviation.

For the comparison of patient backgrounds, patients with severe sepsis/septic shock in each of the two cohorts were further divided into two categories based on IRGM(+313) (rs10065172) genotype: CC+CT and TT (Table 2). No significant difference in age, gender, length of ICU stay, or SOFA score was observed between the two genotype categories in either cohort. A significant difference in APACHE II score between genotype categories was observed only in the discovery cohort: TT homozygotes exhibited a significantly higher APACHE II score than CC+CT genotypes (26.5±8.1 vs. 21.3±8.1, *P = *0.031).

**Table 2:** Severe sepsis/septic shock (SS) patient’s baseline characteristics with regard to IRGM(+313) (rs10065172) genotypes.

	Discovery cohort				Multi-center validation cohort			
	All	CC+CT	TT	*P* value	All	CC+CT	TT	*P* value
	(n = 125)	(n = 112)	(n = 13)		(n = 268)	(n = 242)	(n = 26)	
Age (years), mean±SD	57±17	56.7±17.3	65.1±14.4	0.097*	66±15	67±15	67±15.3	0.861*
Male/female gender, n	69/56	61/51	8/5	0.771**	173/95	155/87	18/8	0.671**
Length of ICU stay (days), mean±SD	18.4±22.0	19.2±22.9	11.7±8.8	0.243*	26.3±35	26.3±35.7	24.6±26	0.811*
SOFA score, mean±SD	10.3±4.6	10.0±4.5	12.5±5.1	0.076*	9.4±4.2	9.4±4.2	10.8±4.1	0.094*
APACHE II score, mean±SD	21.9±8.3	21.3±8.1	26.5±8.1	0.031*	21.8±8.0	21.9±7.9	21.4±8.4	0.758*
Mortality (%)	29.6	26.8	53.8	0.043^#^	25.4	23.6	42.3	0.037^#^

*P* values were calculated with Student’s t-test*, Fisher’s exact test**, and correlation/trend test^#^. SS, severe sepsis/septic shock; ICU, intensive care unit; SOFA, sequential organ failure assessment; APACHE II, the acute physiology and chronic health evaluation; SD, standard deviation.

### 2. Genotype Distributions

Table 3 summarizes the genotype distributions in all subjects of the present study. The subjects were divided into three groups (severe sepsis/septic shock (SS), non-sepsis, and healthy volunteers) for comparison of distribution of the three genotypes (CC, CT, and TT). The genotypic distribution in IRGM(+313) did not diverge from Hardy-Weinberg equilibrium in all the three studied groups. As shown in Table 3, no apparent difference in the percentages of the CC/CT/TT genotypes was observed among the three groups.

**Table 3:** Genotype distributions.

IRGM(+313) genotypes	SS patients	Non-sepsis patients	Healthy volunteers
	n (%)	n (%)	n (%)
CC	164 (41.7)	242 (43.7)	144 (41.5)
CT	190 (48.3)	245 (44.3)	163 (47.0)
TT	39 (9.92)	66 (11.9)	40 (11.5)
Hardy-Weinberg equilibrium test *P* value	0.133	0.741	0.549

SS, severe sepsis/septic shock.

### 3. Comparison of Mortality Rate

**Figure 1:**
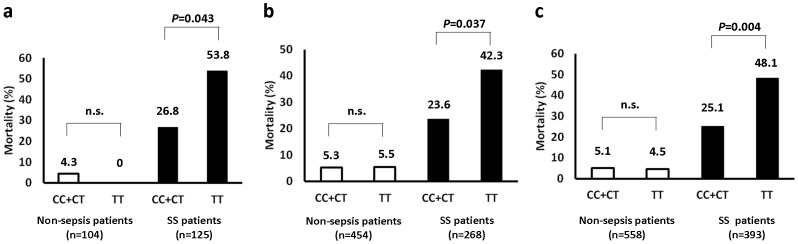
Comparison of mortality between different genotype categories for SNP at IRGM(+313) (rs10065172). (a) The discovery cohort (*P* = 0.043, recessive model of the correlation/trend test; TT v (CC+CT) in the 125 SS patients). (b) The multi-center validation cohort (*P* = 0.037, recessive model the correlation/trend test; TT v (CC+CT) in the 271 SS patients). (c) The combined cohort (*P* = 0.004, recessive model the correlation/trend test; TT v (CC+CT) in the 396 SS patients). SS, severe sepsis/septic shock.

For both of the cohorts, the mortality rate was compared between two different genotype categories separately within the non-sepsis and SS groups. For the discovery cohort, no significant difference in mortality was observed between the CC+CT genotype and TT homozygotes in the non-sepsis group, while TT homozygotes exhibited a significantly higher mortality rate than the CC+CT genotype in the SS group (*P = *0.043) (Table2, Fig. 1a). Similar results were obtained for the multi-center validation cohort (*P = *0.037) (Table2, Fig. 1b).

When the two cohorts were combined and analyzed in a similar manner for validation, the statistical significance of the difference in mortality rate between the two genotype categories was even greater (*P = *0.004) (Fig. 1c). The model treating the C allele as dominant, which combines the CC and CT genotypes into one category, provides the smallest *P* value of all the association tests (Table 4). That is the reason why we performed the further analysis on mRNA expression by comparing TT and CC+CT genotypes.

**Table 4:** Tests of association between IRGM(+313) genotype and severe sepsis death in the combined cohort.

	Genotype			*P* value
	CC	CT	TT	
Additive model OR (vs CC)	1	0.86	2.82	0.126
Dominant T allele OR (vs CC)	1	1.06	1.06	0.803
Dominant C allele OR (vs CC+CT)	1	1	2.63	0.004
Allele table, T vs C allele	1(C)	1.28(T)		0.141

*P* values were calculated with correlation/trend test.

### 4. *Ex vivo* LPS Challenge and mRNA Assay

The genotype distribution in the healthy volunteer group (n = 347) was not apparently different from that in either the SS group or the non-sepsis group (Table 3). When *ex vivo* LPS challenge and mRNA assays were performed for 70 subjects in the healthy volunteer group, the mean ΔRQ for the TT homozygotes (−0.3465, SD = 0.1133) was significantly lower than the values for the CC homozygote (1.427, SD = 0.5809) and the CT heterozygote (0.6702, SD = 0.1702) (*P = *0.019) (Table 5).

**Table 5:** IRGM(+313) mRNA expression of whole blood with LPS challenge in healthy volunteers.

IRGM(+313) genotypes	CC	CT	TT
(n = 70)	(n = 33)	(n = 33)	(n = 4)
Mean ΔRQ	1.427	0.6702	−0.3465
Std. Error	0.5809	0.1702	0.1133

*P* = 0.019 with Mann-Whitney U test ((CC+CT) v TT).

RQ, relative quantification; ΔRQ, (post-stimulation)-(pre-stimulation with LPS).

## Discussion

As described above, we demonstrated that TT homozygosity at the IRGM(+313) (rs10065172) SNP locus, an autophagy-related genetic polymorphism, was associated with a significantly higher mortality rate in SS group patients in two cohorts, a single-center cohort and a multi-center cohort. When the two cohorts were combined and analyzed in a similar manner for validation, the statistical significance of the difference in mortality rate between TT homozygotes and the CC+CT genotype category was even greater. In addition, when whole blood was exposed to stimulus by LPS and *IRGM* mRNA expression was assayed, the amount of mRNA expressed was significantly lower in the TT homozygotes.

The development of organ dysfunction in severe sepsis may be ascribable to the dysfunction or death of cells constituting the deteriorated organ [13]. While cell death induced by cellular dysfunction has traditionally been interpreted as the result of necrosis, a different type of cell death, apoptosis, was identified as an additional mechanism of cell death in sepsis, depending on the cell type [24]. Apoptosis is a mechanism of regulated suicide of cells, *i.e.*, programmed cell death, triggered to protect an individual. In addition to necrosis and apoptosis, autophagy has recently been highlighted as a third mechanism of cell death [7].

Autophagy is a physiological phenomenon of “self-eating” that involves self-degradation of proteins by cells experiencing unfavorable conditions for survival, such as a poor nutritional environment, *i.e.* starvation. Autophagy contributes to the maintenance of biological homeostasis by recycling proteins and eliminating pathogenic microorganisms that invade the cytoplasm [12]. When cells are starved, protein synthesis slows down. Autophagy occurs in such a situation to enable cells to transfer their own amino acids to other cells. During the process of autophagy, cell organelles are incorporated into autophagosomes, which fuse with lysozomes for intracellular digestion. Which cells fall into necrosis, apoptosis, or autophagy under a highly invasive condition such as sepsis remains controversial [7], [9], [11], [25]–[27].

Autophagy has been reported in a number of cases of sepsis. Crouser et al. [9] implicated mitochondrial depletion is related to the removal of damaged mitochondria by autophagy during mouse cecal ligation and puncture (CLP) model of sepsis. Watanabe et al. [11] observed increased numbers of autophagosomes in the liver cells of patients with severe sepsis and further demonstrated increased numbers of autophagosomes in the liver of a mouse CLP model. Using the same animal model of sepsis in GFP-LC3 transgenic mice, Takahashi et al. [28] demonstrated a transient increase in autophagic activity during the acute phase of sepsis with subsequent stagnation of autophagic flux. While the relationship between autophagy and sepsis is beginning to be clarified, the details of their association remain unknown.

IRGM was originally identified as a molecule with an important function in eliminating *Mycobacterium tuberculosis* from the host cell via autophagy [29]. We believe the present work has major implication for the broader field of sepsis because of the similarities between active tuberculosis and proacted sepsis. The possible involvement of *IRGM* polymorphism in the development of Crohn’s disease, an inflammatory bowel disease, has been reported. Work by Brest et al. [30] implicated a variant at the IRGM(+313) locus in the activity of infection control in the intestinal epithelia of individuals with Crohn’s disease. They demonstrated that a family of microRNAs overexpressed under inflammatory conditions formed a complex with *IRGM* mRNA to regulate IRGM production.

*IRGM* polymorphism has also been reported to be involved in mitochondrial dysfunction. Singh et al. [31] showed that IRGM translocates to the mitochondrial inner membrane to regulate mitochondrial nuclear fission. They also reported that the overexpression of a particular IRGM isoform caused mitochondrial nuclear fission and depolarization as well as autophagy-independent cell death. Furthermore, Carre et al. [32] reported that the activation of mitochondrial biogenesis in the skeletal muscle of septic patients contributed to a favorable outcome. Watanabe et al. [11] reported that liver cells from patients with sepsis had both a reproducible pattern of mitochondrial injury and a marked increase in autophagic vacuolization. Takasu et al. [26] identified mitochondrial membrane injury and autophagosome formation in renal proximal tubules of septic patients.

Autophagy and organ failure have recently gained attention in the field of surgical nutrition. Vanhorebeek et al. [33] have reported that a relatively fasted state is required for proper functioning of autophagy in nutrition support during the first days of critical illness. Early overfeeding under a highly invasive condition might lead to the insufficient activation of autophagy. This would lead to susceptibility to infections resulting from defects in host defense mechanisms as well as abnormal activity of the cellular injury repair system. Consequently, the accumulation of various injuries at the cellular level would induce organ dysfunction and delay recovery. These undesirable factors would ultimately affect each other. We hypothesized that the ‘physiologic’ autophagy flux is disturbed in severely septic conditions and this impaired autophagy causes the patient deterioration through tissue dysoxia. The less expresser in *IRGM* SNP, *i.e.*, TT homozygotes of IRGM(+313), might cause the harmful effect in the pathophysiology of severe sepsis/septic shock.

The present study has the following limitations: First, the pathogen for sepsis was identified in only a limited number of septic patients included in the study. Although the mechanisms of autophagy against different pathogenic microorganisms such as *Mycobacterium tuberculosis*, *Listeria*, and *Toxoplasma* are being clarified separately, the pathogen was unknown in the majority of our septic patients. Consequently, the mechanism of autophagy in each septic patient also remains unknown and must be discussed only with respect to sepsis in general.

Second, the patients studied were admitted to the participating centers over a period of 12 years although they were included without any overlaps. The standard treatment for severe sepsis/septic shock might not be identical throughout such a long study period, and the possible influence of treatment variation on clinical outcome of the patients cannot be ruled out. Third, the *IRGM* and the gene product might be associated with the role of autophagy in bacterial processing, however data that correlates this with bacterial clearance has not been demonstrated. We speculate that this impaired autophagic response that is altered by the TT homozygote lead to decreased *IRGM* expression, hampered the autophagic function of limiting cellular and organ injury [34]. Finally, the concentration of LPS used in LPS challenge of whole blood collected from healthy humans (final concentration, 1 ng/mL) was much higher than that used for LPS challenge in animal studies. Regardless of the limitation, a significant suppression of IRGM gene expression at the mRNA level in TT variant homozygotes in response to LPS in human whole blood cells was observed for the first time in this study. The assay of *IRGM* mRNA in blood collected from patients with severe sepsis for comparison among different genetic variants may be of significance in elucidating the genetic mechanisms regulating autophagy. However, this approach may not always be easy, since the expression of various genes related to the inflammatory response is known to change during the clinical course of sepsis and vary depending on the etiology [35].

## Conclusions

In the present single-center and multi-center study, we demonstrated that TT homozygosity at the IRGM(+313) locus, an autophagy-related polymorphic locus, affected clinical outcome in patients with severe sepsis. LPS challenge of whole blood from healthy volunteers showed significantly reduced expression of *IRGM* in TT homozygotes. The present study suggests the possibility that TT homozygosity at the IRGM(+313) locus may be involved in the suppression of *IRGM* expression in severe sepsis, thereby suppressing autophagy and leading to a poor clinical outcome.
